# Reversible pH Responsive Bovine Serum Albumin Hydrogel Sponge Nanolayer

**DOI:** 10.3389/fbioe.2020.00573

**Published:** 2020-06-03

**Authors:** Vikram Singh Raghuwanshi, Brendan Yu, Christine Browne, Gil Garnier

**Affiliations:** Bioresource Processing Research Institute of Australia (BioPRIA), Department of Chemical Engineering, Monash University, Clayton, VIC, Australia

**Keywords:** bovine serum albumin (BSA), adsorption, pH, QCM-D, gold, AFM, dynamic light scattering (DLS), contact angle

## Abstract

A pH dependent reversible sponge like behavior of a bovine serum albumin (BSA) nanolayer adsorbed at the gold-saline interface is revealed by quartz crystal microbalance with dissipation (QCM-D), atomic force microscope (AFM) and contact angle measurements. During the saline rinsing cycles, the BSA layer adsorbs water molecules at pH 7.0 and releases them at pH 4.5. The phenomenon remains constant and reproducible upon multiple rinsing cycles. The BSA layer thickness also increases upon rinsing with saline at pH 7.0 and reverses back to its original thickness at pH 4.5. Varying ionic strength with water further desorbs more water molecules from the BSA layer, which decreases its mass and thickness. However, upon both pH and ionic strength changes, all the BSA molecules remain adsorbed irreversibly at the gold interface and only the sorption of water molecules occurs. The study aims at engineering high efficiency pH-responsive biodiagnostics and drug delivery systems.

## Graphical Abstract 1

Schematic of pH dependent reversible sponge BSA nanolayer at the gold interface.

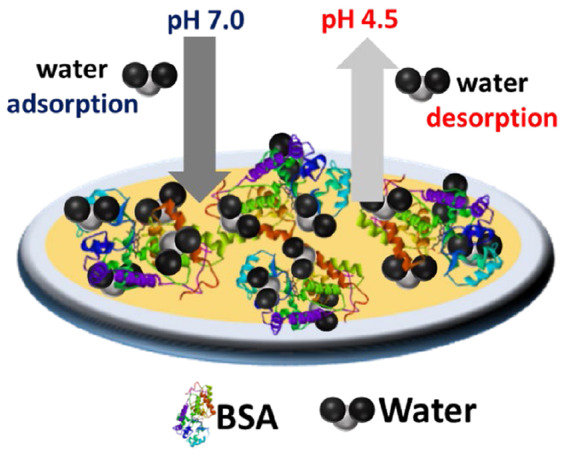

## Introduction

Serum albumins are proteins commonly used in bio-diagnostics and as model in bio-interface research (Rosi and Mirkin, 2005; Singh et al., 2005; Arcot et al., 2015). Among those, bovine serum albumin (BSA) is the cheapest and a protein widely used as blocking agent in ELISA tests (Maingonnat et al., 1999). In paper diagnostics, BSA (Huang et al., 2018) selectively increases paper hydrophobicity to improve bio-fluids and elution flow by decreasing liquid absorption. BSA protects and increases the lifetime of functional biomolecules dried on paper. The functionality and longevity of immunoglobin G and immunoglobin M dried on BSA treated surfaces can increase by an order of magnitude (van Remoortere et al., 2001). BSA also prevents unspecific adsorption of analytes proteins for quantitative analysis.

Multiple articles have extensively reported on the BSA molecules sorption phenomenon at different interfaces such as gold (Dennison et al., 2017), mica (Fitzpatrick et al., 1992), silicon (Jachimska et al., 2016; Givens et al., 2017), and cellulose (Mohan et al., 2014; Lombardo et al., 2017). The adsorbed BSA molecule’s conformation and the topology of the formed layer are strongly affected by pH, ionic strength and temperature. BSA molecules retain their native structure between pH 4.0 and 8.0. Below pH 4.0 and above 8.0, BSA molecules change their folding conformation which differs from their native structure (Su et al., 1998a; Barbosa et al., 2010; Phan et al., 2015). The isoelectric point of BSA is at pH 4.5. At this pH, the net surface charge becomes zero and BSA molecules aggregate. Increasing pH increases the BSA charge and the dominating electrostatic repulsion stabilizes BSA molecules and prevents aggregation (Li et al., 2008).

In spite of being among the most studied proteins, multiple questions remain on the effect of pH and ionic strength have on BSA conformation upon adsorption. In this context, the concept of surface coverage, solely defined by surface fraction or weight density, lacks clarity. There is a need to better understand the variables defining the BSA solid-liquid interface to engineer robust bio-diagnostic devices.

Bovine serum albumin molecules adsorb at an interface forming a layer with a thickness in the nanometer scale. A few characterization methods such as reflectivity (Su et al., 1998b, 2016; Raghuwanshi et al., 2017a, b), ellipsometer, atomic force microscope (AFM), surface plasmon resonance (SPR) and quartz crystal microbalance with dissipation (QCM-D) can measure the adsorbed protein layer thickness at the nanometer scale required. In particular, QCM-D can kinetically monitor the biomolecules sorption process by measuring the adsorbed protein mass at an interface in nanograms (Kristensen et al., 2013; Luan et al., 2017). QCM-D enables the control of temperature, ionic strength and pH environment. The dissipation mode of QCM reveals the rigidity of the adsorbed protein layers.

In this study, a reversible pH responsive behavior of BSA molecules adsorbed at the gold-saline interface is described. The adsorbed BSA layer behaves as a pH sensitive sponge where the water molecules adsorb and desorb depending on the surrounding pH between 4 and 8. This work monitors and quantifies the water sorption phenomenon in BSA sponge like layer and elucidates the mechanisms involved at different pH and ionic strength. It is our objective to describe the BSA coverage at the solid-liquid interface in terms of number of molecules and weight/thickness of the layer. This is to clarify the concept of biomolecule surface coverage and to elucidate the dynamic behavior of adsorbed BSA molecules in the context of bio-diagnostics.

## Materials and Experiments

### Materials

Bovine Serum Albumin lyophilized powder (97%) and sodium chloride (NaCl) salt (99.5%) were purchased from the Sigma Aldrich (Castle Hill, NSW, Australia). Hydrochloric acid (HCl) and sodium hydroxide (NaOH) were purchased from Merck Ltd. All chemicals are analytical grade and used without any purification.

### QCM-D Measurements

Quartz crystal microbalance with dissipation measurements were performed on an E4-QCM-D instrument from Biolin Scientific Ltd. Gold coated quartz crystal sensors were used after cleaning in a H_2_O_2_:NH_3_:H_2_O (1:5:5) solution for 15 min and followed by UV-Ozone cleaning for 10 min.

The gold sensors were placed in liquid cell modules. 1 mg/mL BSA was dissolved in the saline solution (0.9% NaCl) and the pH of the solution was set to pH 7.0 and 4.5. Separately, the saline solution pH was adjusted to 7.0 and 4.5. The prepared solutions were passed through the liquid cell modules by a peristaltic pump. Changes in the quartz sensor resonance frequency (F) and dissipation (D) with respect to the fundamental frequency of 5 MHz and six different odd overtones (1, 3, 5, 7, 9, and 13) were simultaneously monitored.

First a saline solution was pumped in the liquid cell and allowed to equilibrate to generate a stable baseline. Afterward, BSA in saline solution was passed through the cell, and allow the BSA molecules to adsorb at the gold interface. Saline was then pumped to remove any non-attached BSA molecules. Rinsing cycles of salines at different pH and water was then as follow:

A) Mode 1: Saline pH 7.0 (Baseline)→ BSA/Saline pH 7.0 (Absorption)→ Rinse with Saline pH 7.0→ Rinse Saline pH 4.5→ Rinse Water→ Rinse Saline 4.5→ Rinse Saline 7.0
B) Mode 2: Saline pH 4.5 (Baseline)→ BSA/Saline pH 4.5 (Absorption)→ Rinse with Saline pH 4.5→ Rinse Saline pH 7.0→ Rinse Saline 4.5→ Rinse Water→ Rinse Saline 4.5→ Rinse Saline 7.0

The obtained variations in the resonance frequency ΔF and the dissipation ΔD were fitted by the Sauerbrey model by using the software Dfind.

### DLS Measurements

Dynamic light scattering (DLS) on the BSA dispersed in the saline solution at different pH (4.5 and 7.0) were measured on DLS particle size analyzer (Brookhaven Nanobrook Omni). A source of 40 mW (640 nm) temperature-controlled red semiconductor laser was used. Measurements were performed three times and averaged. All measurements were conducted at the room temperature (22°C).

### Contact Angle Measurements

Contact angle on gold and BSA adsorbed at different pH on a gold interface were measured using a setup OCA 35 DataPhysics Instruments GmbH, Germany. Measurements were conducted directly on the sensor surface taken out of the QCM setup after measurement. All measurements were performed at room temperature (22°C). A minimum of five contact angle measurements were performed on the sensor surface and averaged.

### Atomic Force Microscope (AFM)

Atomic force microscopy measurements were conducted in tapping mode with a JPK Nanowizard III AFM. The cantilevers (AC160TS-R3) selected for imaging had a nominal frequency of 300 kHz and spring constant of 26 N/m. The imaging was performed on the bare gold interface and the adsorbed BSA layer at pH 4.5 at the gold interface. The images were taken directly on the sensor surface taken out of the QCM setup after measurement. All measurements were performed at the room temperature (22°C).

## Results

A reversible pH responsive water sorption phenomenon of BSA molecules adsorbed at the solid-liquid interface is studied by QCM-D with rinsing cycles of saline solution at pH 7.0 and 4.5. Gold was selected as solid interface as its hydrophobicity directs BSA adsorption (Lori and Hanawa, 2004; Phan et al., 2015; Ozboyaci et al., 2016). The BSA layers adsorbed at different pH values are rinsed with alternate cycles of saline solutions at pH 4.5 and 7.0. In addition, rinsing with pure Milli-Q water was conducted to evaluate the effect of ionic strength has on the adsorbed BSA layer.

**FIGURE 1:**
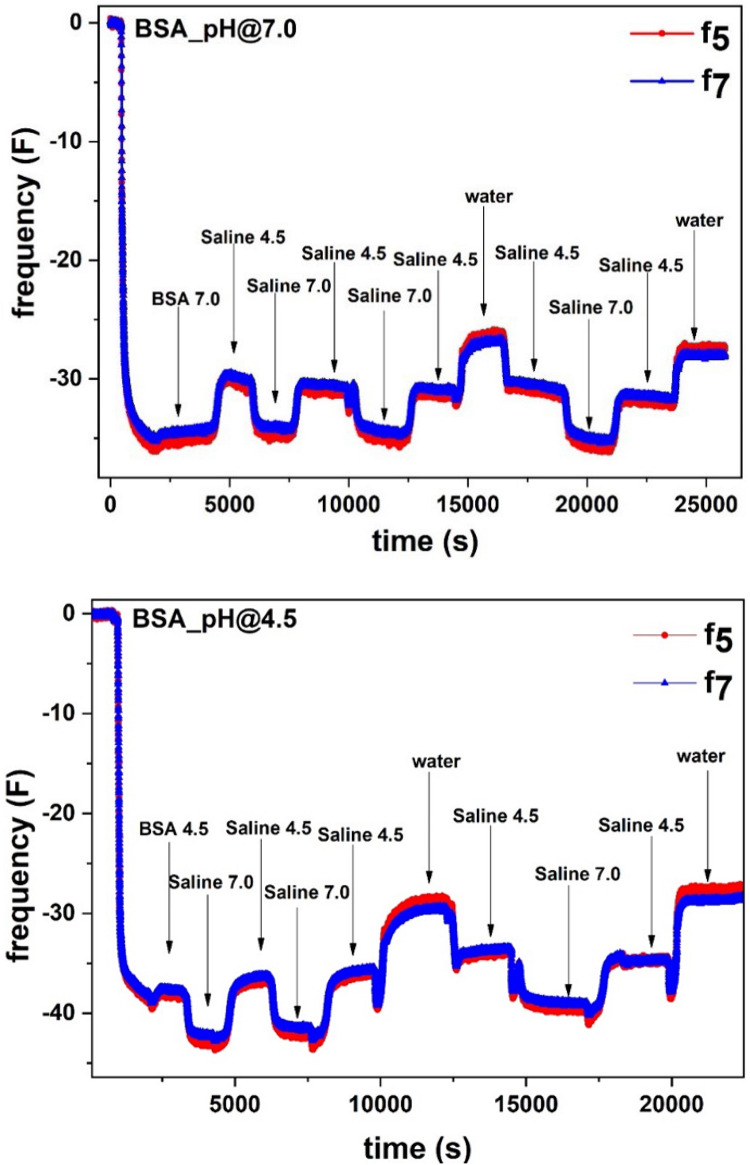
**(Top)** Adsorption of BSA (1 mg/mL) in 0.9% NaCl saline solution at pH 7.0 on the liquid-gold interface. After BSA adsorption saturation, the sensor surface was rinsed with the saline solution at pH 7.0, followed by rinsing cycles of saline at pH 4.5, pH 7.0 and water. **(Bottom)** Adsorption of BSA (1 mg/mL) in 0.9% NaCl saline solution at pH 4.5 on the liquid-gold interface. After BSA adsorption saturation, the sensor surface was rinsed with the saline solution at pH 4.5, followed by rinsing cycles of saline at pH 7.0, pH 4.5 and water.

Figure 1 (Top) shows the change in frequency (F_5_ and F_7_) for the BSA molecules adsorbed at 7.0 pH from the BSA/saline solution followed by rinsing with the original saline solution (pH 7). The next rinsing cycle was performed with the saline solution at pH 4.5. Alternate cycles of saline solutions at pH 4.5 and 7 then follow.

In Figure 1, after an initial stable baseline, a sudden decrease in F was observed which indicates BSA molecules adsorption at the gold-liquid interface. The F decreased up to ΔF = −35.5 and stabilized. Upon rinsing with saline (pH 7), the F increased from ΔF = −35.5 to ΔF = −34.0 which shows removal of the non-adsorbed BSA molecules from the surface. Following rinsing with saline (pH 4.5), the F further increased to ΔF = −30.0, revealing additional mass decay from the sensor surface. Surprisingly, later saline (pH 7.0) rinsing cycles decrease F to ΔF = −34.0, which means an increase in mass at the sensor surface due to water molecules absorption in the BSA layer. Subsequent saline rinsing cycles follow the same cyclic mass change at the gold interface.

In the second experiment, similar to the first experiment, BSA molecules adsorption at pH 4.5 was followed by saline rinsing cycles at different pH (Figure 1: Bottom). The adsorbed BSA molecules correspond to the decrease in F to ΔF = −38.5. Rinsing with saline (pH 4.5) removes the non-adsorbed BSA (ΔF = −38.0).

Following rinsing by saline (pH 7.0) further increases the mass of the layer at the gold surface which corresponds to the decrease in F to ΔF = −43. The increase in mass is due to the absorption of water molecules in the BSA layer. Later, saline (pH 4.5) rinsing desorbs water molecules and brings back the *F* value to ΔF = −37. Each rinsing cycle adsorbs and desorbs the same amount of water molecules.

In the same experiment, the effect of ionic strength on the adsorbed BSA layer was studied by rinsing the layer with pure water. Figure 1 shows the BSA adsorption at pH 7.0 and 4.5 followed by rinsing cycles with saline at different pH and with pure Milli-Q water.

In both cases, water rinsing increases the value in ΔF = −29.2 (BSA adsorbed at pH 4.5) and −26.5 (BSA adsorbed at pH 7.0). This indicates that rinsing with water further decreases the mass which corresponds to further desorption of water molecules from the interface. Each rinsing cycle sustains the same behavior in the mass change which is due to the water sorption in the BSA layer.

Interestingly, in all experiments, alternating saline rinsing cycles at pH 4.5 and 7.0 show the reversible sorption of water molecules within the adsorbed BSA layer. Rinsing the BSA layer with saline at pH 7.0 adsorbs water molecules within the BSA layer structure which increases the mass of the solid-liquid interface. In contrast, rinsing the BSA layer with saline at pH 4.5 desorbs water molecules from the BSA layer which reduces the mass of the interface. The fully reversible water sorption measured indicates the BSA molecules do not desorb during rinsing and their surface coverage remains identical; only the number of water molecules in the interphase varies.

The water sorption phenomenon upon saline rinsing (at different pH) occurs only due to adsorbed BSA layer and is confirmed by a separate saline rinsing experiment on the bare gold sensor (Supplementary Material S1). A stable baseline on the bare gold sensor frequency is maintained by the saline solution (at pH 4.5). Afterward, the gold interface was rinsed with alternative saline rinsing cycle of pH 7.0 and 4.5 (Supplementary Material S1). Results clearly show that the alternative saline rinsing at different pH has no effect on the gold sensor frequency. Therefore, only the adsorbed BSA layer on gold exhibits the change in frequency on saline rinsing cycles at different pH values.

The adsorbed mass, the surface coverage and the thickness of the adsorbed BSA layer are extracted by fitting the Sauerbrey model to the QCM data. The model is used to fit a rigid layer where the dissipation value is less than 2, as observed in all our experiments (Supplementary Material S2). The Sauerbrey equation is given by $\Delta \,m=-\frac{C\,\Delta \,f}{n}$, where, C = 17.7 ng/Hz.cm^2^ is constant for the 5 MHz gold coated quartz crystal, *n* is the overtone, Δ*m* is the adsorbed mass and Δ*f* is the change in frequency.

**FIGURE 2:**
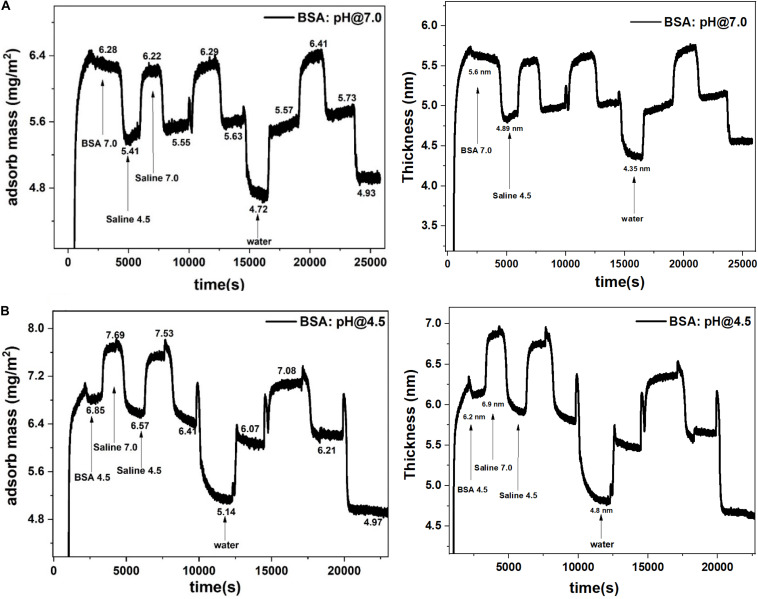
Adsorbed BSA mass (left) and thickness (right) at the gold interface and changes with the saline rinsing cycles at pH 7.0 and 4.5. **(A)** BSA adsorbed at pH 7.0 and rinsed. **(B)** BSA adsorbed at pH 4.5 and rinsed.

The BSA molecules adsorbed up to a mass coverage of 6.3 mg/m^2^ (thickness 5.6 nm) at pH 7.0 (Figure 2A). Rinsing pre-adsorbed BSA layer with saline (pH 4.5) decreased the mass coverage to 5.6 mg/m^2^ and its thickness to 4.9 nm (Table 1), which is due to the release of water molecules from the adsorbed BSA layer structure. Further rinsing with saline (at pH 7.0) re-adsorbs water molecules at the same amount. The mass change difference is Δm = 0.7 mg/m^2^.

**TABLE 1:** Adsorbed mass (mg/m^2^) from modeling the QCM-D data with the Sauerbrey model.

**pH**	**Adsorb water mg/m^2^**	**Desorb water mg/m^2^**	**Mass difference ΔM**	**Sorbed water molecules/m^2^ (×10^19^)**	**Water molecules sorbed/BSA molecule**
7.0	6.3	5.6	0.7	2.3	450
4.5	7.4	6.4	1.0	3.3	570

Similarly in Figure 2B, rinsing the pre-adsorbed BSA layer at pH 4.5 with saline (at pH 7.0) increases the mass adsorbed from 6.4 mg/m^2^ to 7.4 mg/m^2^ and the thickness from 6.2 to 6.9 nm; this is due to water molecules absorption in the BSA layer. The rinsing cycle of saline at different pH values kept the mass change difference of Δm = 1.0 mg/m^2^ which is 1.4 times higher than the mass change at pH 7.0 (0.7 mg/m^2^).

The average number of water molecules adsorbed/desorbed in the BSA layer during saline rinsing cycle is calculated from the difference in mass adsorbed at different pH (Supplementary Material S3). The BSA layer adsorbed at pH 4.5 adsorbs/desorbs 3.3 × 10^19^ water molecules during the rinsing cycles, which represents 570 water molecules/BSA molecule (Table 1). However, the BSA layer adsorbed at pH 7.0, adsorbs/desorbs 2.3 × 10^19^ water molecules, or 450 water molecules/BSA molecule, during the reversible saline rinsing cycle.

**FIGURE 3:**
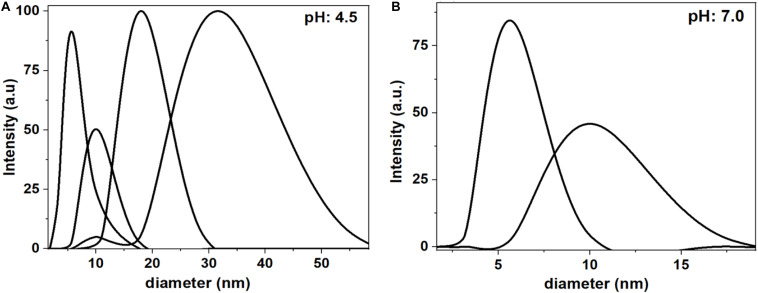
Dynamic Light Scattering (DLS) measurements of BSA in saline at pH 4.5 **(A)** and pH 7 **(B)**. At pH 4.5, the BSA shows multiple size distribution with the maximum at 5, 10, 20, and 50 nm. At pH 7.0, the BSA shows only two size distributions at 5 and 10 nm.

Dynamic Light Scattering (DLS) measurements clarify the aggregated and non-aggregated state of BSA in saline at pH 4.5 and pH 7.0 (Figure 3). At pH 4.5, DLS reveals the BSA molecules aggregates and shows multiple size distributions: 5 nm, 10 nm, 20 nm and 50 nm. However, at pH 7.0, the BSA molecules do not aggregate, due to electrostatic repulsions, and have size distribution of 5 and 10 nm. The 5 and 10 nm size of hydrated BSA is comparable to the size and shape of individual BSA molecules (14 nm × 4 nm × 4 nm) (Anderegg et al., 1955; Wright and Thompson, 1975).

**FIGURE 4:**
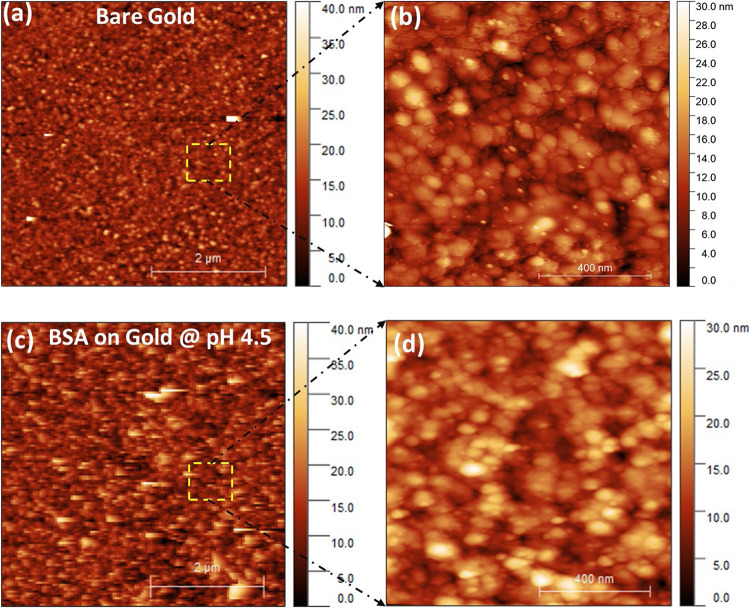
**(a)** AFM images of the bare gold interface, **(b)** Enlarged/magnified image of bare gold interface, **(c)** BSA layer adsorbed at the gold interface at pH 4.5 **(d)** Enlarged/magnified image of BSA coated gold.

Atomic force microscope images confirm the adsorption of BSA molecule at the gold interface (Figure 4). These images demonstrate differences in the surface morphology of bare gold (Figures 4a,b) and the BSA adsorbed at the gold interface at pH 4.5 (Figures 4c,d). Upon comparison of the enlarged images of the bare gold (Figure 4b) and BSA absorbed surface (Figure 4d) the differences between the surfaces is noticeable. Even though both surfaces have particles forming them, the definition and therefore the material imaged is different. The particles of the bare gold surface are more defined (e.g., sharper boundaries between shapes) which indicates a harder material when compared to the BSA coated surface. The BSA coated gold shows the presence of additional aggregates of BSA molecules. The enlarged AFM image of BSA coated gold (Figure 4d) shows the lateral dimension of the aggregated BSA molecules varies between 30 and 100 nm with height in the range of 5–15 nm.

**FIGURE 5:**
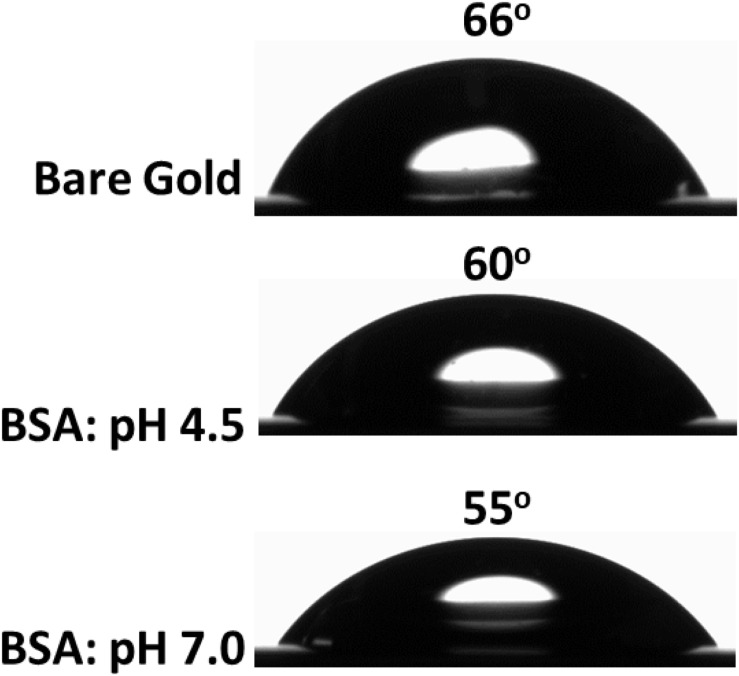
Contact angle measurement of the bare gold interface **(top)** and the BSA layer adsorbed at the gold interface at pH 4.5 **(middle)** and at pH 7.0 **(bottom)**.

The contact angle formed by water droplets on two surfaces: gold and BSA adsorbed on gold was measured to clarify wettability (Figure 5). The gold sensor is hydrophilic with a contact angle of 66°. However, the BSA layer adsorbed at pH 4.5 becomes more hydrophilic as the water contact angle decreased to 60°, which further decreased to 55° for the BSA layer adsorbed at pH 7.0. A similar observation was reported for BSA layers adsorbed onto a silicon surface as the water contact angle decreased from 57° (at pH 4.5) to 54° (at pH 7.0) (Jachimska et al., 2016). Change in the contact angle and layer thickness for BSA adsorbed at different pH indicate structural and topography differences during the adsorption process at the gold interface.

## Discussion

The BSA isoelectric point is between pH 4.5–4.8; it is the pH at which the molecule net charge becomes zero. Near the isoelectric point, the BSA molecules have less inter-molecule electrostatic repulsion. The high ionic strength of saline (0.15 M) also plays a role in screening charges and hindering electrostatic interactions. Therefore, BSA molecules aggregate in the BSA/saline suspension. DLS measurements (Figure 3A) confirm the presence of BSA aggregates of sizes up to 60 nm in the BSA/saline suspension at pH 4.5.

**FIGURE 6:**
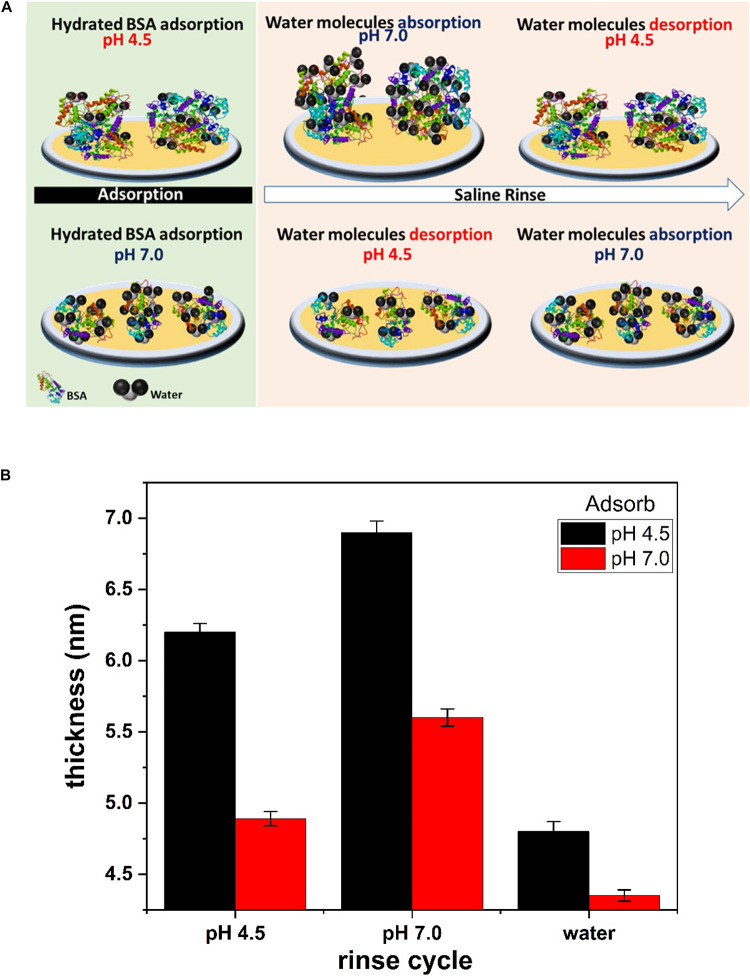
**(A)** Schematic representation of BSA sorption and conformation on the liquid/gold interface at pH 4.5 and 7.0. Saline rinse of BSA layer adsorb/desorb water molecules at pH 7.0/4.5. **(B)** Thickness of the BSA layer evaluated from the Sauerbrey model. The hydrated BSA layer adsorbed at pH 4.5 and pH 7.0 is plotted at different saline and water rinse cycle.

During BSA adsorption (at pH 4.5) at the gold interface, no electrostatic attraction of BSA toward the gold interface is expected. However, a weak positive charge from the globular BSA protein can provide a sufficient drift for adsorption at an interface (Su et al., 1998a; Jachimska et al., 2016). Multiple articles have previously reported the adsorption of BSA and similar proteins near the isoelectric point to be driven by hydrophobic interactions which outweigh the electrostatic interactions (Uyen et al., 1990; Tilton et al., 1991; Figueira and Jones, 2008; Norde, 2008; Jeyachandran et al., 2009; Rabe et al., 2011; Huang et al., 2017; Xu et al., 2018; Attwood et al., 2019). The contact angle measurements (Figure 5) show the bare gold interface is less hydrophobic, and albumin binds with gold via hydrophobic interactions (Norde and Giacomelli, 2000; Figueira and Jones, 2008). Since the inter-BSA molecules repulsions are screened, the hydrated BSA molecules adsorb in large amounts (6.4 mg/m^2^) as aggregates and with multiple contact points onto the gold interface (Figure 6A). The AFM image (Figures 4c,d) confirms the adsorption and aggregation of BSA molecules at the gold interface. AFM images reveal the lateral dimension of aggregates ranges between 30 and 100 nm with the height distributed between 5 and 15 nm. This confirms the BSA molecules adsorbed as a combination of both standing and flat conformations.

At pH 7.0, the BSA molecules are negatively charged. This creates an electrostatic repulsion between BSA molecules which hinders BSA agglomeration in solution. DLS measurements show the distribution of non-aggregated BSA molecules of size 5 and 10 nm (Figure 3B). These sizes are comparable with the dimensions of individual BSA molecules (14 nm × 4 nm × 4 nm) (Anderegg et al., 1955; Wright and Thompson, 1975). During BSA adsorption on gold, a combination of both electrostatic repulsion and hydrophobic interactions form a BSA layer at the interface. The strong lateral inter-molecule repulsion between the adsorbed BSA molecules reduces the BSA adsorption capacity (5.6 mg/m^2^) at the interface. Therefore, BSA molecules adsorb as individual molecules (not aggregates) and form a monolayer at the gold interface (Figure 6A).

In the QCM-D experiments (Figure 1), pre-hydrated BSA molecules are adsorbed at the interface. Upon alternative saline rinsing at different pH, the BSA layer further adsorbs/desorbs water molecules. The BSA adsorbed at pH 4.5 entraps and releases more water molecules (1.0 mg/m^2^) compared to the BSA molecules adsorbed at pH 7.0 (0.7 mg/m^2^). The reason is the amount of BSA adsorbed at pH 4.5 (6.4 mg/m^2^) is larger than that at pH 7.0 (5.6 mg/m^2^).

The calculated dry mass (non-hydrated) of the BSA molecules adsorbed for complete surface coverage of the gold sensor is about 2 mg/m^2^ (Supplementary Material S3). The calculated dry mass is comparable with the reported literature (Jachimska et al., 2016). When a dry BSA molecule is hydrated, its hydrophilic groups bound with water rapidly. The binding is due to the water dipolar structure interacting with the polar groups in BSA. In hydrated BSA, some water molecules are bound firmly, while other water molecules are bound loosely or are simply entrapped between the loop structure of BSA. The amount of water hydrating the BSA layer increases the adsorbed mass fraction at the interface. In the saline rinsing cycle at different pH values lead to a redistribution of charges on the adsorbed BSA layer. This charge redistribution creates a gradient between the adsorbed BSA layer and the bulk solution. The gradient acts as a driving force to entrap and release loosely bound water molecules from the BSA layer.

The thickness of the BSA layer is evaluated by fitting the Sauerbrey model to the QCM-D data (Figure 2). The hydrated BSA layer adsorbed at pH 4.5 and rinsed with saline (pH 7.0) gives the larger thickness of 6.9 nm (Figure 6B). The large amount of adsorbed BSA molecules (6.4 mg/m^2^) entraps many water molecules which swells the BSA layer. Rinsing the same layer with the saline (pH 4.5) reduces the layer thickness to 6.4 nm, which is due to the release of water molecules from the layer. A similar phenomenon is observed for the BSA molecules adsorbed at pH 7.0. However, the BSA layer thickness is thinner than for the BSA layer adsorbed at pH 4.5 (Figure 6B).

Further, the adsorbed BSA layer at both pH values remains rigid and irreversibly attached during saline rinsing cycles. Only sorption of water molecules occurs during pH variation. The adsorbed BSA rigidity and irreversibility is due to the BSA large size and high molecular weight. The BSA molecule forms a multitude of contact points at the gold-liquid interface by electrostatic and hydrophobic interactions which prevent desorption of BSA molecules from the interface.

The BSA layer does not detach from the gold interface even with changes in solution ionic strength (rinsing with deionized water). Water rinsing only desorbs more water molecules from the BSA layer and the mass on the sensor surface further decreases (Figure 2). Changing the ionic strength of hydrated BSA with pure water releases more water molecules from the layer. The BSA layer shrinks to a thickness 4.8 nm (when adsorbed at pH 4.5) and 4.3 nm (when adsorbed at pH 7.0) as shown Figure 6B. Continuing saline rinsing cycles produces the same reversible water sorption phenomenon.

## Conclusion

Bovine serum albumin in saline solution (0.9% NaCl) was adsorbed at the gold-liquid interface at pH 7.0 and 4.5. The dynamic process was measured by QCM-D and confirmed by AFM, DLS and contact angle measurements. A reversible, fast and pH dependent water sorption phenomenon is observed for the adsorbed BSA layer by performing rinsing cycles of saline at pH 4.5 and 7.0. The water molecules hydrate the BSA layer at pH 7.0 and dehydrate it at pH 4.5. The BSA layer adsorbed at pH 4.5 is hydrated by 1.4 times more water molecules than the BSA layer adsorbed at pH 7.0. This phenomenon is explained by the different conformations adopted by BSA molecules adsorbed at different pH. Near the isoelectric point at pH 4.5, the BSA molecules neutralize and adsorb as aggregates in large amounts: 6.4 mg/m^2^. At pH 7.0, the BSA molecules become charged (electrostatic repulsion) and adsorb as a layer of individual molecules at 5.6 mg/m^2^. The layer of aggregated BSA molecules (at pH 4.5) adsorbed at the gold interface entraps more water molecules (570 water molecules/BSA) than the layer of individual BSA molecules (at pH 7.0), which retain 450 water molecules/BSA. Changing the ionic strength by rinsing the BSA layer with pure water only desorbs more water from the adsorbed layer structure. In all cases, the BSA layer is rigid and irreversibly adsorbed onto the gold interface and only the water molecules adsorb/desorb during the rinsing cycle. The observed phenomenon is important for fundamental understanding and to engineer novel bio-diagnostic devices and robust sensors.

## Data Availability Statement

The raw data supporting the conclusions of this article will be made available by the authors, without undue reservation, to any qualified researcher.

## Author Contributions

VR, CB, and BY conducted the experiments. VR and GG performed the data analysis and wrote the manuscript.

## Conflict of Interest

The authors declare that the research was conducted in the absence of any commercial or financial relationships that could be construed as a potential conflict of interest.
