# Supplementary material for: Reversible pH Responsive Bovine Serum Albumin Hydrogel Sponge Nanolayer
Source: Front Bioeng Biotechnol. 2020 Jun 3;8:573. doi: 10.3389/fbioe.2020.00573 (PMC7286146; doi:10.3389/fbioe.2020.00573)
Supplement: Supplementary file 1 [file Data_Sheet_1.pdf]

Supporting Information

Reversible pH Responsive Bovine Serum Albumin Hydrogel Sponge Nanolayer

Vikram Singh Raghuwanshi, Brendan Yu, Christine Browne, Gil Garnier

Bioresource Processing Research Institute of Australia (BioPRIA),Department of Chemical Engineering, Monash University, Clayton,Victoria, Australia

S1: Bare gold coated quartz sensor rinsed with the saline at different pH of 4.5 and 7.0.

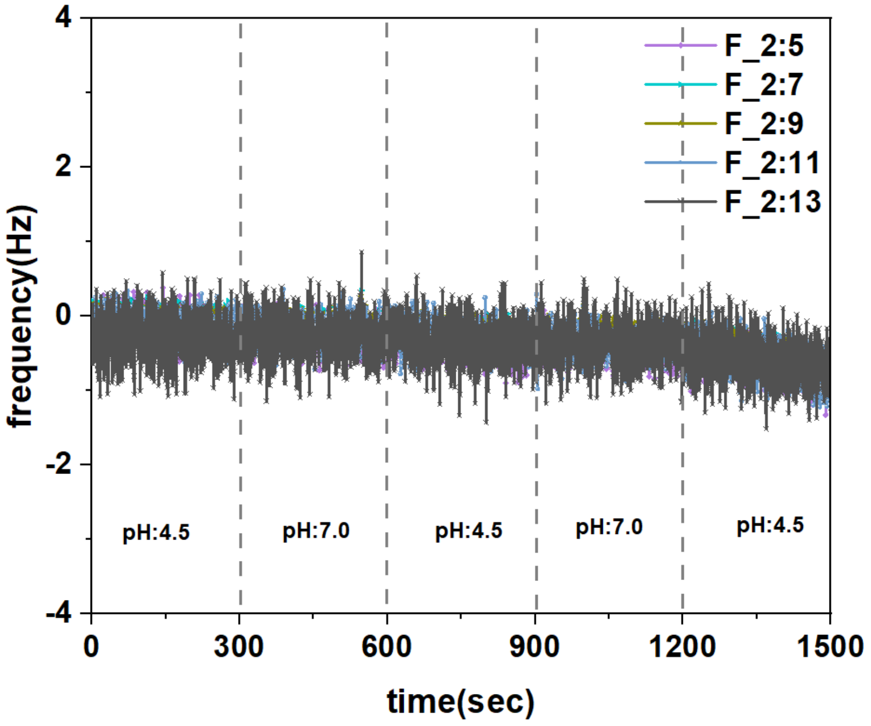

S2: Dissipation of BSA adsorbed at pH 4.5 and 7.0 followed by saline rinse cycle at different pH.

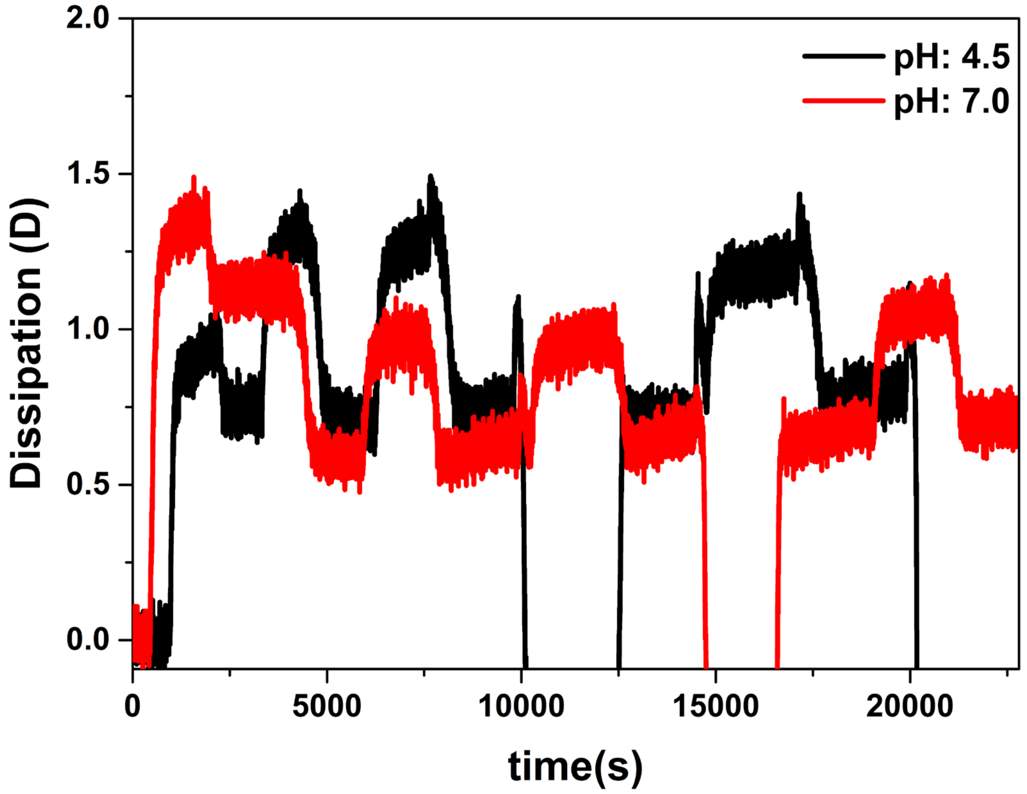

S3: Calculation on BSA sorption at the gold interface

Calculated number of BSA molecules and adsorbed water per BSA molecule

Calculated number of BSA molecules and adsorbed water per BSA molecule

BSA in 0.9% NaCl saline solution was made at a concentration of 1 mg/mL. The mass of a single BSA molecule is 1.1 x 10^{-16} mg (dry mass) and that of a single water molecule is 2.99 x 10^{-20} mg.

The number of BSA molecules from the wet mass of BSA (6.4 mg/m^2) at the sensor surface is calculated at pH 4.5 as:

6.4/1.1 x 10^{-16} = 5.82 x 10^{16} BSA molecules/m^2

The number of adsorbed water molecules is evaluated from the difference of the mass with and without water molecules (1.0 mg/m^2):

1.0/2.99 x 10^{-20} = 3.3 x 10^{19} adsorbed water molecules/m^2

This provides the number of water molecules attached/trapped per BSA molecule and is calculated as:

Number of water molecules/BSA = 3.3 x 10^{19} /5.8 x 10^{16} = **569 or 570**

Similarly, the number of BSA molecules at the sensor surface is calculated at pH 7.0 as:

5.6/1.1 x 10^{-16} = 5.1 x 10^{16} BSA molecules/m^2

Number of Water molecules = 0.7/2.99 x 10^{-20} = 2.3 x 10^{19} adsorbed water molecules

Number of water molecules/BSA = 2.3 x 10^{19} /5.1 x 10^{16} = **450**

Relative number of BSA molecules adsorbed (dry mass) at the sensor

BSA molecules have dimensions of 14 nm x 4 nm x 4 nm and modelling of the QCM-D data gives an average thickness of 5.5 ± 0.5 nm. Therefore, it is likely that the BSA molecule retains in a lying conformation at the gold interface and forms a rigid monolayer (5 nm thick). Assuming that BSA molecules in the lying position cover the complete surface of the sensor, then the number of BSA molecules adsorbed is:

Surface area of sensor of 1 cm in diameter = π x 0.5^2 = 0.8 cm^2

Surface area of a BSA molecule in a flay lying conformation = 14 nm x 4 nm = 56 x 10^{-14} cm^2

Therefore, the number of BSA molecule covering the complete surface of sensor is:

0.8/56 x 10^{-14} = **1.4 x 10^{12} molecules**

Or BSA molecules/m^2 = 1.4 x 10^{12}/0.8 x 10^{-4} =1.75 x 10^{16} molecules/m^2

The weight of a BSA molecules determined as:

BSA molecular weight: 66400 g/mol

1 mole of BSA = 66400 g

6.023 x 10^{23} molecules of BSA = 66400 g

1 molecules of BSA = 1.1 x 10^{-19} g or 1.1 x 10^{-16} mg

The total mass of adsorbed BSA on sensor surface (1 cm in diameter) is:

1.1 x 10^{-19} g x 1.4 x 10^{12} molecules = 1.5 x 10^{-7} g OR **0.15 µg**

The BSA mass adsorb per unit area of sensor:

0.15 x 10^{-3} mg / 0.8 x 10^{-4} m^2 = **1.9 mg/m^2**
